# Systematic Analysis to Identify the MIR99AHG-has-miR-21-5p-*EHD1* CeRNA Regulatory Network as Potential Biomarkers in Lung Cancer

**DOI:** 10.7150/jca.93343

**Published:** 2024-03-04

**Authors:** Mengju He, Hui Zhang, Yanfei Zhang, Yicen Ding, Fei Zhang, Yani Kang

**Affiliations:** 1 School of Biomedical Engineering, Bio-ID Center, Shanghai Jiao Tong University, Shanghai, 200240, China.; 2 Shanghai Lung Cancer Center, Shanghai Chest Hospital, Shanghai Jiao Tong University School of Medicine, Shanghai,200030, China.; 3 Shanghai Starriver Bilingual School, Shanghai, 201108, China.

**Keywords:** Lung cancer, LncRNA-miRNA-mRNA regulatory network, Systematic analysis, Potential biomarkers

## Abstract

Lung cancer (LC) remains an extremely lethal disease worldwide, and effective prognostic biomarkers are at top priority. With the rapid development of high-throughput sequencing and bioinformatic analysis methods, the quest to characterize cancer transcriptomes continues to move forward. However, the integrated systematic analysis of lncRNA-miRNA-mRNA regulatory network in LC is lacking. In this study, we collected samples of cancer tissues and adjacent normal tissues from patients with lung cancer and conducted transcriptome and small RNA sequencing to identify differentially expressed genes (DEGs), miRNAs (DEMs), and lncRNAs (DELs). The regulatory roles of miRNAs in LC were explained by functional analysis on DEM-targeted genes. The lncRNA-miRNA pairs, miRNA-mRNA pairs, and lncRNA-mRNA pairs were identified and combined to construct the interplay of lncRNA-miRNA-mRNA. We evaluated the prognostic value of selected lncRNA-miRNA-mRNA by Kaplan-Meier analysis. Finally, we analyzed the expression levels of selected DEM, DELs, and DEGs in lung cancer patients and healthy people to verify our findings. A total of 1492 DEGs, 12 DEMs, and 604 DELs were identified in LC patients. Based on the bioinformatic analysis and the regulatory mechanism of ceRNAs, 3 lncRNAs (GATA2-AS1, LINC00632, MIR99AHG), 1 miRNA (hsa-miR-21-5p) and 5 targeted genes (*RECK*, *TIMP3*, *EHD1*, *RASGRP1* and *ERG*) were figured out first. Through further Kaplan-Meier analysis screening the prognostic value, we finally found the hub subnetwork (MIR99AHG-hsa-miR-21-5p-*EHD1*) by collating lncRNA-miRNA pairs, miRNA-mRNA pairs and lncRNA-mRNA pairs. As the key of ceRNA regulatory network, the expression of miRNA-21-5p in lung cancer patients was significantly higher than that in healthy people (*P* < 0.01), and its high expression was significantly associated with poor prognosis (*P* = 0.0025). Our study successfully constructed a MIR99AHG-hsa-miR-21-5p-*EHD1* mutually regulatory network, suggesting the potential efficient biomarkers in LC.

## Introduction

Lung cancer (LC) is the leading cause of cancer-attributable deaths worldwide, accounting for 21% of cancer deaths and 1.8 million deaths each year 1, 2. LC can be classified into two main sub-groups according to their biological characteristics, non-small cell lung cancer (NSCLC) and small cell lung cancer (SCLC), which account for 84% and 13% respectively 1, 3, 4. Since LC has few initial symptoms, figuring out efficient biomarkers and the mechanism of LC is a matter of some urgency for prognosis and treatment.

Non-coding RNAs (ncRNAs) are a class of non-protein-coding transcripts, regulating gene expression through interaction with others in the transcriptional and posttranscriptional stages 5. Therein, microRNAs (miRNAs) and long non-coding RNAs (lncRNAs) are the main two types. MiRNAs are a sort of ~22-nucleotide small ncRNAs, which induce mRNAs degradation or translation repression by completely or non-completely binding to the sequences of the targeted mRNAs in the 3'untranslated region (3'UTR) 6. MiRNAs have been extensively studied as biomarkers in cancer diagnosis and prognosis 7, 8. Additionally, lncRNAs, with lengths over 200 nucleotides, have been identified for the regulatory function 9. Different from the intensively studied miRNAs, the potential values of lncRNAs were accumulating over the past few years. LncRNAs are involved in promoting chromatin modification, managing protein synthesis, inducing mRNA decay and tumorigenesis 10-12.

Accumulated studies showed the interactions between miRNAs and lncRNAs. They can act not only as protagonists on the other, but also act in combination to influence other biological processes. MiRNAs can lead to miRNA-triggered lncRNA decay, lncRNAs can act as miRNA sponges and generate miRNAs, and the combination of the both can serve as competing endogenous RNAs (ceRNAs) 13, 14. For instance, upregulated lncRNA SNHG1 targets miR-101-3p, which activates the Wnt/β-catenin signaling pathway to influence the progression of NSCLC 15. The interaction of LINC01140 and miR-377-3p/miR-155-5p has an effect on the proliferation, migration, invasion, and immune escape of LC cells 16. Many studies on ceRNA only focused on miRNAs and explored the interaction between lncRNA-miRNA and miRNA-mRNA, because miRNAs play a very important role in the ceRNA network. While, according to previous studies, lncRNAs orchestrate the regulation from DNA to RNA to protein and function in transcriptional, epigenetic regulations and posttranscriptional regulation by direct complementary binding to mRNA 17, 18. These evidences suggested that the relationship between lncRNA-miRNA-mRNA and subsequent biological effects are complex. However, the integrated systematic analysis of their interaction in LC remains unclear.

In this study, samples of cancer tissues and adjacent normal tissues from patients with lung cancer were collected and conducted transcriptome and small RNA sequencing. Differentially expressed genes (DEGs), miRNAs (DEMs), and lncRNAs (DELs) in LC tissues and paired normal tissues were comprehensively analyzed by bioinformatical analysis. Then the ceRNA network containing 3 lncRNAs (GATA2-AS1, LINC00632 and MIR99AHG), 1 miRNA (has-miR-21-5p) and 5 targeted genes (*RECK*, *TIMP3*, *EHD1*, *RASGRP1* and *ERG*) was constructed to reveal the regulatory interaction between lncRNA-miRNA-mRNA and figure out potential biomarkers for LC. Finally, through further Kaplan-Meier analysis screening the prognostic value, we found the hub subnetwork (MIR99AHG-has-miR-21-5p-*EHD1*) by combining lncRNA-miRNA pairs, miRNA-mRNA pairs and lncRNA-mRNA pairs. The expression levels of selected DEM, DELs, and DEGs in lung cancer patients and healthy people were analyzed to verify our findings. This study aims to provide a theoretical basis for digging LC pathogenesis and effective prognostic methods from the perspective of the lncRNA-miRNA-mRNA regulatory network based on our own transcriptome sequencing data.

## Materials and Methods

### Clinical samples collection

Lung cancer tissues and paired normal tissues from three patients with LC were collected at Shanghai Chest Hospital. This study was approved by the Institutional Review Boards of Shanghai Jiao Tong University School of Medicine and chest hospital Ethics Committee (KS(Y)1987). This study was carried out in accordance with the Code of Ethics of the World Medical Association (Declaration of Helsinki). All patients participating in this study signed an informed consent form. LC clinical samples, after being isolated from organs, were quickly put into liquid nitrogen and stored at -80°C for long-term preservation.

### Total RNA extraction

Lung cancer tissues and paired normal tissues, after being taken out from -80°C refrigerator, were ground with liquid nitrogen and the temperature was required to keep as low as possible. Then, TRIzol reagents (Invitrogen, Waltham, MA, USA) were added and tissues were ground sufficiently. Finally, the homogenates were transferred to EP tubes and the total RNA was extracted following the manufacturer's protocol. The total RNA quantity was determined with Nanodrop 2000 (Thermo Scientific, Waltham, Massachusetts, USA).

### Transcriptome sequencing and preprocessing

The RNA-seq libraries were constructed using the KAPA Stranded RNA-Seq Library Preparation Kit (KAPA Biosystem, Wilmington, MA, USA). Then the quality of libraries was assessed by Agilent 2100 Bioanalyzer (Agilent, USA) to ensure that they met the requirements of sequencing. The quality of raw sequencing data of RNA-seq was first assessed using FastQC (version 0.11.9). Then sequencing reads were mapped to the Homo sapiens genome assembly GRCh38 using Hisat2 (version 2.2.1) after reads filtering. The read counts were calculated using stringtie (version 1.3.3) and merged into a matrix. Then data of mRNA and lncRNA were separated based on the annotation from ENSEMBL. Differential expression analysis was performed on mRNA data using edgeR (version 3.42.4) and DESeq2 (version 1.40.2) and on lncRNA data using DESeq2 (version 1.40.2), with *P* < 0.05 and |log_2_FC| > 1 considered as statistically significant. The analysis results of DEseq2 and edgeR were intersected as differentially expressed genes (DEGs). ClusterProfiler (version 4.8.3) package was used for Gene Ontology (GO) and Kyoto Encyclopedia of Genes and Genomes (KEGG) analysis to explore the possible mechanism of these genes involved in the occurrence and development of lung cancer. GO terms were divided into three classifications, including molecular functions (MF), cellular components (CC) and biological processes (BP). Using *P* < 0.01 as the criterion, we screened the ten most significantly enriched functional terms in each category for visualization.

### Small RNA sequencing and data analysis

The small RNA-seq libraries were constructed using TruSeq Small RNA Library Prep Kit (NEB, San Diego, California, USA). Then the quality was also assessed by Agilent 2100 Bioanalyzer (Agilent, USA) with the total RNA in the same way. After the quality inspection passed, the next generation sequencing was carried out. Sequencing data of miRNA were first assessed using FastQC (version 0.11.9) and then preprocessed with cutadapt (version 3.5). Then paired-end reads were merged into single-end reads using fastq-join in ea-utils tool (version 1.7.1). Clean reads were then aligned to the human reference genome (hg38) using bowtie (version 1.0.0) and counted using htseq (version 0.13.5). Finally, DESeq2 (version 1.40.2) and limma (version 3.56.2) were used to identify differentially expressed miRNAs (DEMs), at a cutoff of *P* < 0.05 and |log_2_FC| > 1. Raw data of transcriptome sequencing and small RNA sequencing were submitted to the GEO database, and the accession number referred to GSE194302.

### Integrative analysis of the lncRNA-miRNA-mRNA regulatory relationship

Targeted genes of DEMs were predicted through TargetScan database (version 8.0) 19. GO and KEGG functional analyses were conducted to explore the function of targeted genes involved, with *P* < 0.01 considered as statistically significant. We obtained the miRNA-mRNA interactions via miRNA target prediction. Then the intersections of prediction results and differentially expressed genes (DEGs) were preserved for further analysis. The lncRNA-miRNA interactions were found in LncBase v3, a module of DIANA-Tools. The results were refined by a set of filter options, including direct validation type and high miRNA confidence level. To better understand the complexity of ceRNA regulatory network, we predicted the lncRNA-mRNA interactions using LncRRIsearch, based on the calculation of local base-pairing interaction energy to predict the complementary binding of lncRNA to mRNA 20. Finally, lncRNA-miRNA-mRNA regulatory relationships were integratedly analyzed.

### Kaplan-Meier analysis

To evaluate the prognostic value of the selected RNAs and figure out the subnetwork, we assessed the identified 3 lncRNAs (GATA2-AS1, LINC00632, MIR99AHG), 1 miRNA (hsa-miR-21-5p) and 5 genes (*RECK, TIMP3, EHD1, RASGRP1* and* ERG*). A matrix of miRNA and lncRNA expression data and associated clinical information for lung adenocarcinoma (LUAD) was obtained from The Cancer Genome Atlas (TCGA) database using the R package TCGA-biolinks (version 2.14.0). 567 samples of LUAD data, including 521 primary cancer tissue samples and 46 normal samples, each measuring 1881 miRNAs and 12,727 lncRNAs, were used for survival analysis. Only tumor samples were used for survival analysis, and after integration of the clinical information with the expression matrix, 521 tumor samples were obtained for miRNA survival analysis and 488 tumor samples for lncRNA survival analysis.

Survival analysis was performed using survival (version 3.5.7) for the combined matrix of miRNA expression data and clinical information, using median expression data to classify lung cancer patient samples into those with low or high miRNA expression, and log-rank analysis to find *P*-values for each miRNA and plot Kaplan-Meier curves, which were drawn to assess the effect of miRNA on survival time and clinical survival data. Survival analysis using lncRNA expression matrix was performed with the same method. Meanwhile, survival analysis of mRNAs was performed and the overall survival (OS) was evaluated by GEPIA2. Clinical survival data from LUAD and LUSC patients were used for survival analysis.

### Validation of the aberrant expression of selected lncRNA, miRNA and mRNAs

The expression data of hsa-miR-21-5p in lung cancer tissues and normal lung tissues were obtained from OncoMir Cancer Database (OMCD) 21, which provides respectively 458 lung adenocarcinoma (LUAD) data sets and 46 normal data sets, 343 lung squamous cell carcinoma (LUSC) data sets and 45 normal data sets. These two types of lung cancer account for about 85% of all lung cancers. We normalized the miRNA expression data by taking the logarithm after adding 1 to each data. After obtaining the standardized data, we investigated the difference of hsa-miR-21-5p expression levels between lung cancer and normal tissues. LncExpDB was used to verify the lncRNA MIR99AHG expression level in LC and normal tissues achieved by the same standardized processing as for miRNA hsa-miR-21-5p 22. Gene Expression Profiling Interactive Analysis 2 (GEPIA2) was then adopted for mRNAs analysis by using RNA sequencing data, which containing 483 LUAD and 486 squamous cell carcinoma (LUSC) sample data points, and matched TCGA normal tissues. In addition, GEPIA2 also included the sequencing data of tissue samples donated by healthy individuals from GTEx (Genotype-Tissue Expression) 23, which effectively made up for the problem of too few normal tissue samples compared with cancer tissues in TCGA. The box plots were generated with *P* < 0.01 and |Log_2_FC| > 1 as cut-off values 24.

### Quantitative reverse transcription polymerase chain reaction (RT-qPCR)

To validate the results of our analysis, RT-qPCR experiments were performed to verify the expression levels of the key miRNA has-miR-21-5p. We obtained three other pairs of lung cancer tissue and adjacent normal tissue samples for validation experiments. Tissues were ground and RNA was extracted using TRIzol reagents (Invitrogen, Waltham, MA, USA). Then miRNA was reverse transcribed using Takara RR037A reverse transcription kit (Takara, Otsu, Japan) and stem-loop primer. After that, the reverse transcription products were taken for qPCR using Thermo Fisher PowerUp SYBR qPCR Master Mix (Thermo Scientific, Waltham, Massachusetts, USA) following the manufacturer's protocol. U6 was used as the internal control, and the primers are listed in Table S8.

### Statistical analysis

Statistical background correction, normality standardization, and expression level calculation were performed to make the collected data comparable using R software and Excel. The visualization of the expression level was produced by R software and Excel. The ceRNA regulatory network was built by Cytoscape.

## Results

### Identification of DEGs and functional enrichment analysis

**Figure 1:**
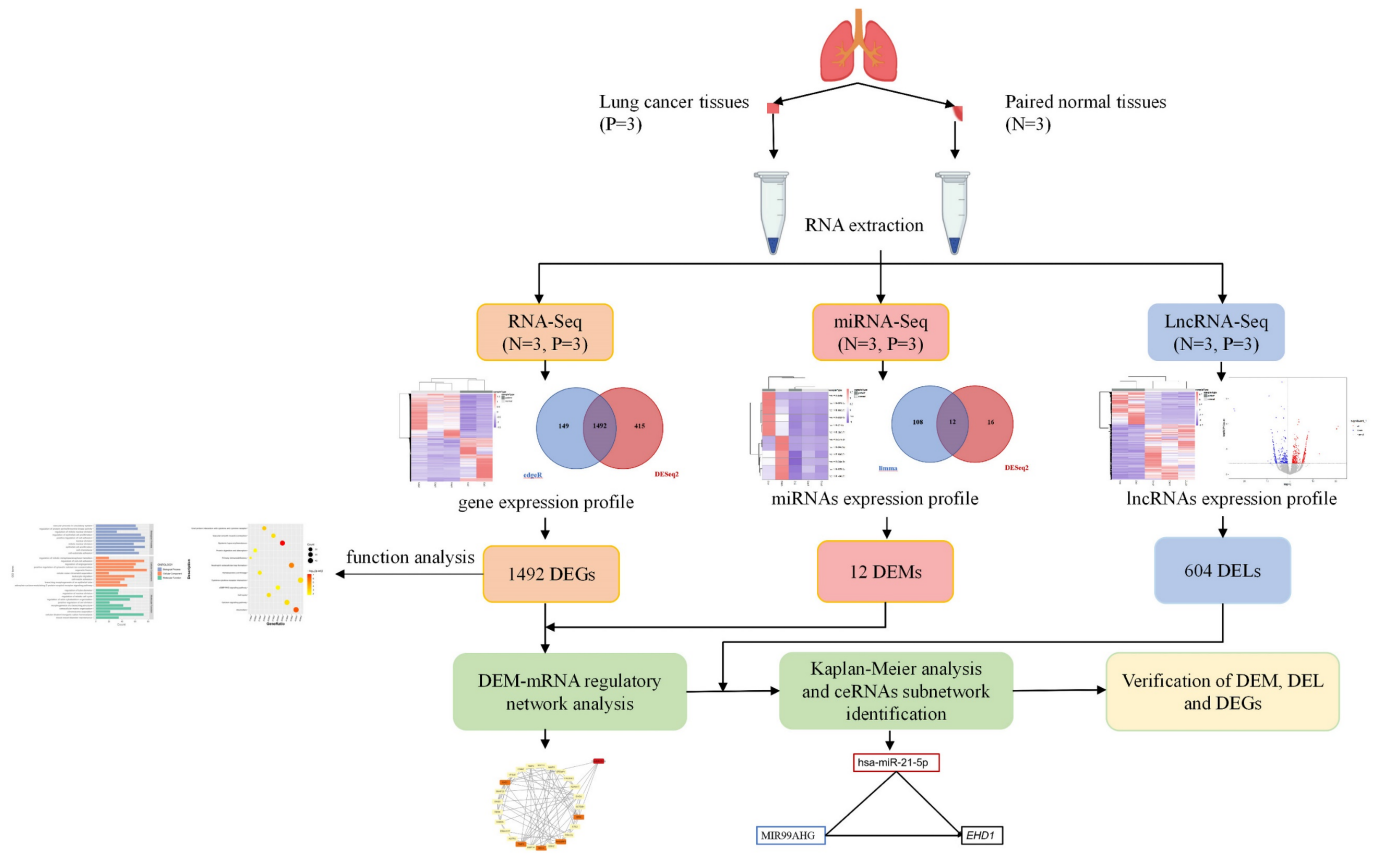
** Graphic workflow of this study.** We collected samples of cancer tissues and adjacent normal tissues from patients with lung cancer and conducted transcriptome and small RNA sequencing to identify differentially expressed genes (DEGs), miRNAs (DEMs), and lncRNAs (DELs). The lncRNA-miRNA pairs, miRNA-mRNA pairs, and lncRNA-mRNA pairs were identified and combined to construct the interplay of lncRNA-miRNA-mRNA. We evaluated the prognostic value of selected lncRNA-miRNA-mRNA by Kaplan-Meier analysis. Finally, we analyzed the expression levels of selected DEM, DELs, and DEGs in lung cancer patients and healthy people to verify our findings.

We used the workflow shown in Figure 1 to complete the entire study. After obtaining RNA-seq and miRNA-seq sequencing data of three pairs of cancer and its adjacent normal tissues, we conducted quality control and analysis of the sequencing data. We found that the sequencing quality of one of the cancer tissues did not meet the requirements. In order not to affect the results of data analysis, subsequent difference analysis was based on two cancerous tissues and three adjacent normal tissues.

**Figure 2:**
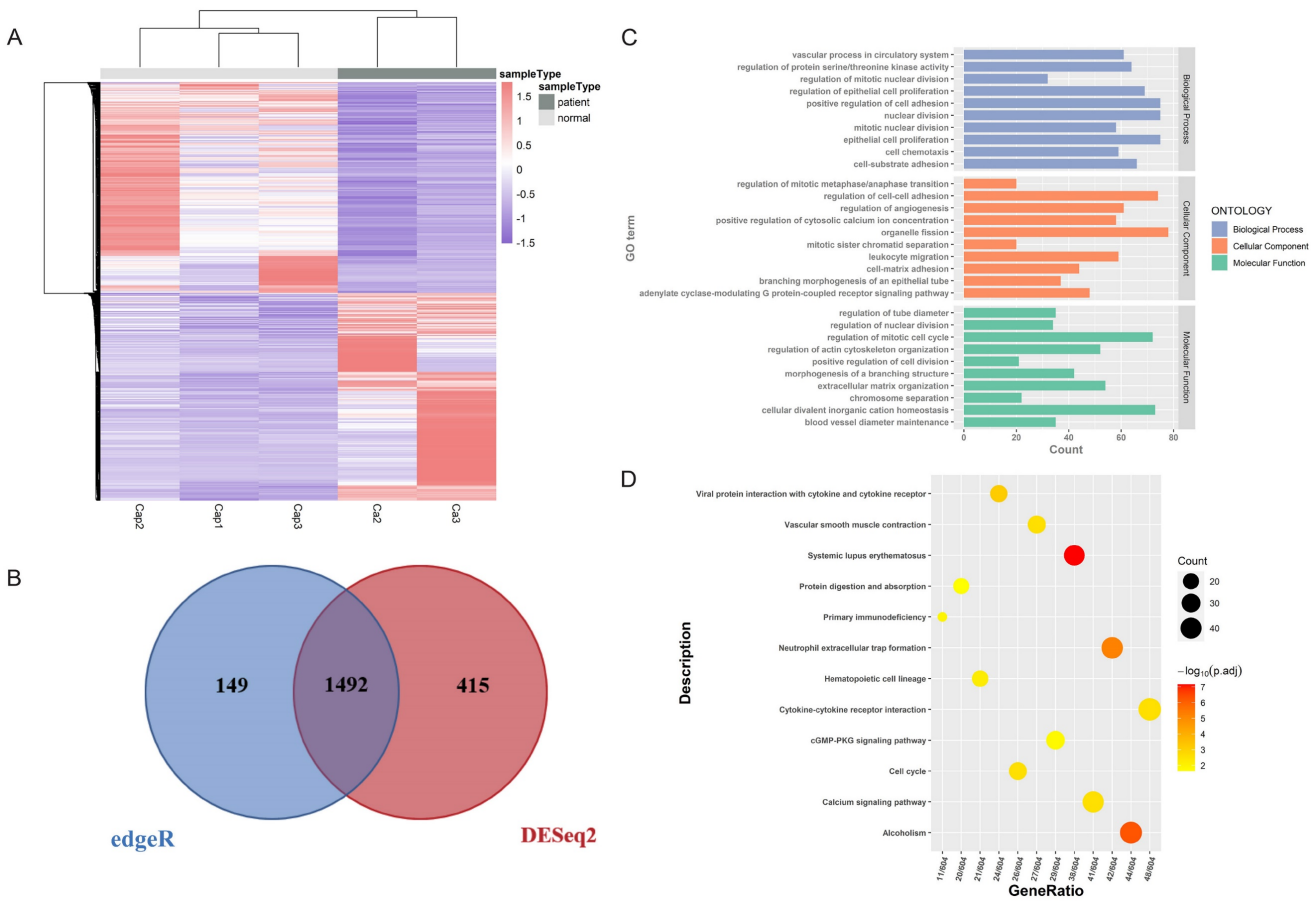
** Identification of differentially expressed genes (DEGs) of tissue samples.** (A) The hierarchical clustering heat map of DEGs. Ca represents cancerous tissue and Cap represents adjacent normal tissue. The heat map shows distinct differences between the groups. (B) The Venn map of DEGs screened out by DESeq2 and edgeR. A total of 1492 DEGs were screened. (C) GO analysis of DEGs (*P* < 0.01). The horizontal axis is the number of genes enriched on each GO term, with different colors representing different functional categories. (D) KEGG analysis of DEGs (*P* < 0.01). Dots of different sizes and colors are used to represent the number of genes enriched on a particular pathway and the significance.

Two methods (edgeR and DESeq2) were used to identify the DEGs accurately. We found 1492 common DEGs, including 752 upregulated genes and 740 downregulated genes (Figure 2A, 2B, Table S1). Significantly distinguished expression patterns were illustrated by the hierarchical clustering analysis and presented in the heatmap (Figure 2A). Then functional enrichment analysis was performed to analyze the regulatory function of DEGs. As shown in Figure 2C and 2D, DEGs were found enriched in several GO terms significantly, including nuclear division (GO:0000280), organelle fission (GO:0048285), regulation of mitotic cell cycle (GO:0007346). Furthermore, Kyoto Encyclopedia of Genes and Genomes (KEGG) enrichment analysis revealed that DEGs were enriched in cytokine-cytokine receptor interaction, neutrophil extracellular trap formation and cGMP-PKG signaling pathway, which were involved in inflammation progression. Functional analysis showed that the differentially expressed mRNAs were mainly involved in the regulation of cell growth and differentiation, and had a certain relationship with inflammation.

### Identification of DEMs and DELs and the lncRNA-miRNA pairs

**Figure 3:**
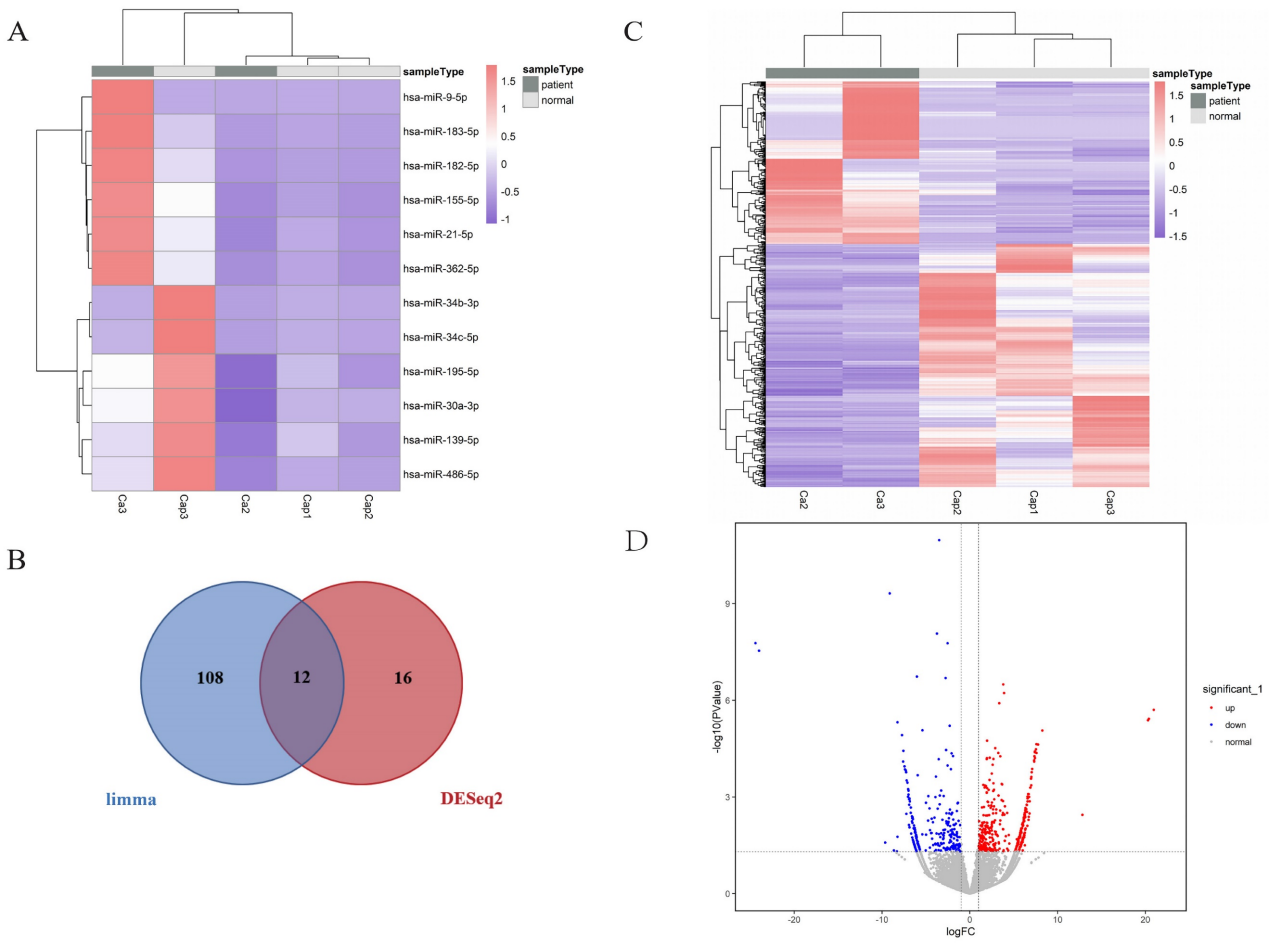
** Identification of differentially expressed miRNAs (DEMs) and differentially expressed lncRNAs (DELs) of tissue samples.** (A) The hierarchical clustering heat map of DEMs in LC and normal tissue samples. Ca represents cancerous tissue and Cap represents adjacent normal tissue. (B) The Venn map of DEMs screened out by limma and DESeq2. A total of 12 DEMs were screened. (C)The hierarchical clustering heat map of DELs. The heat map shows distinct differences between the groups. (D) The volcano map of DELs (*P* < 0.05 and |log_2_FC| > 1). A total of 604 lncRNAs were differentially expressed in cancer tissue and normal tissue samples, of which 362 were high-expressed in cancer tissue (red) and 242 were low-expressed in cancer tissue (blue).

A total of 12 DEMs were identified (Table S2), which were intersections of the results of DESeq2 and limma. Among these DEMs, 6 were upregulated and 6 were downregulated (Figure 3A, 3B). Then 604 DELs were also identified using DESeq2 (Figure 3C, 3D, Table S3). Hierarchical clustering analysis revealed the different expression patterns between LC and normal tissues. By comparing the expression heatmaps of DEGs, DELs and DEMs (Figure 2A, Figure 3A, 3C), we found that the expression profiles of mRNA and lncRNA in cancer tissues and normal tissues showed obvious differences between groups, but the expression profiles of miRNA were not very obvious between groups, which may be due to the small number of screened miRNAs. The interactions between DEMs and DELs were obtained from DIANA-LncBase v3.0, a reference repository with miRNA targets on non-coding transcripts. Finally, 1 DEM, has-miR-21-5p, with its 16 corresponding DELs were identified as miRNA-lncRNA pairs (Table S4).

### Identification of miRNA-mRNA pairs and function analysis of DEM-targeted mRNAs

**Figure 4:**
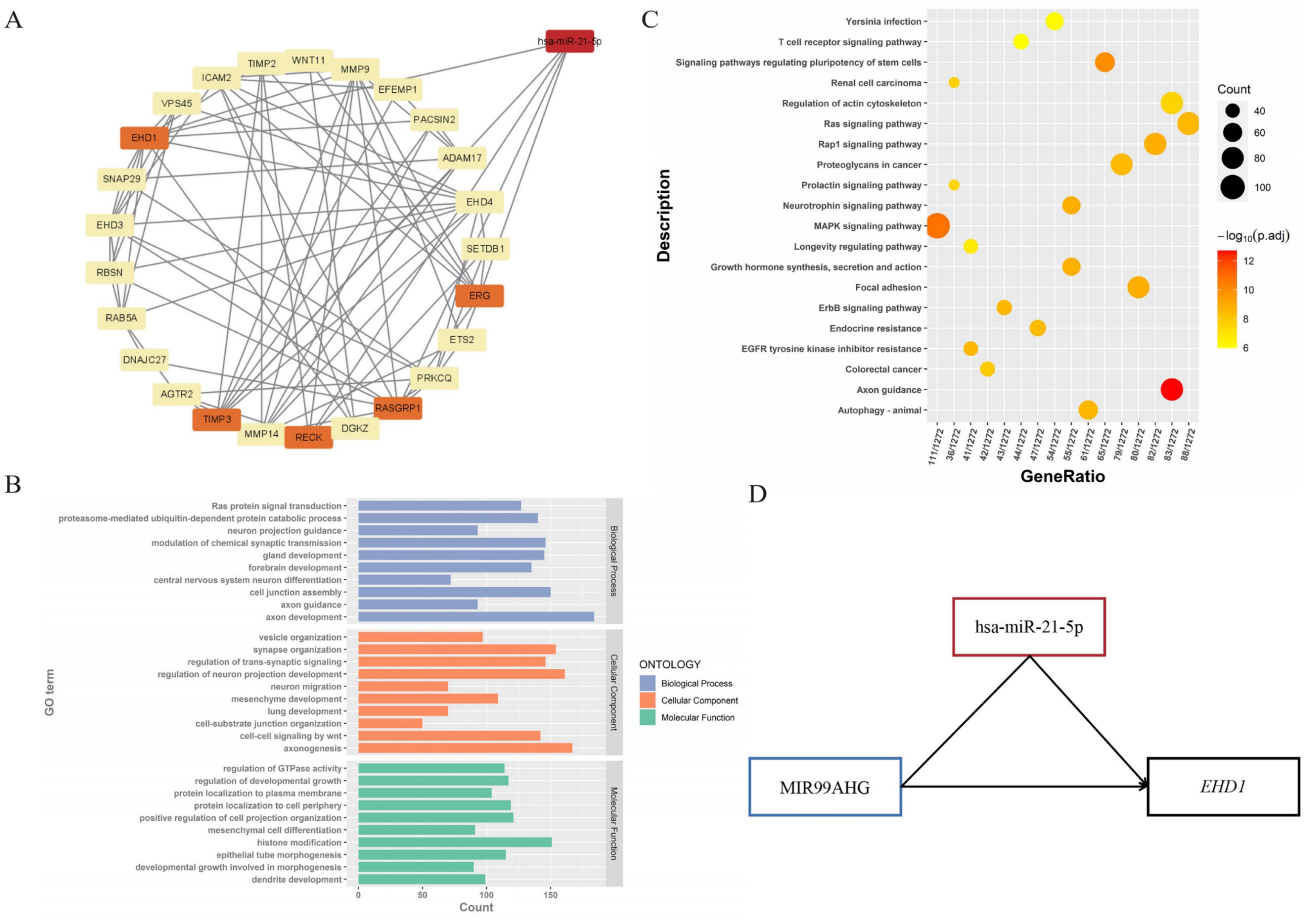
** miRNA-lncRNA-mRNA regulatory network and functional enrichment analysis of DEM-targeted mRNAs.** (A) The constructed miRNA-mRNA network. Hsa-miR-21-5p was screened through lncRNA-miRNA interaction, and its 5 targeted genes (*RECK*, *TIMP3*, *EHD1*, *RASGRP1* and *ERG*) were highlighted in the figure. (B) GO analysis of DEM-targeted mRNAs (*P* < 0.01). The horizontal axis is the number of genes enriched on each GO term, with different colors representing different functional categories. (C) KEGG analysis of DEM-targeted mRNAs (*P* < 0.01). Dots of different sizes and colors are used to represent the number of genes enriched on a particular pathway and the significance. (D) The constructed ceRNAs hub subnetwork. MIR99AHG could emerge as a regulator to play a role in malignant progression via hsa-miR-21-5p-*EHD1* axis in LC, while it also had direct base-pairing interaction with *EHD1*.

To explore the regulatory functions between DEMs and their targeted genes, DEM-DEG interaction pairs were identified (Table S5). The top one miRNA hsa-miR-21-5p and 5 targeted genes (*RECK*, *TIMP3*, *EHD1*, *RASGRP1* and *ERG*) were shown in Table S4. The regulatory network was constructed using Cytoscape 25 (Figure 4A). The functional enrichment analysis revealed that DEM-targeted mRNAs were significantly enriched in longevity signaling pathway, rap1 signaling pathway, proteoglycans in cancer (KEGG pathways) and lung development, ras protein signal transduction and primary epithelial tube morphogenesis (Figure 4B, 4C). These results suggested that the selected miRNA was involved in the development and progression of lung cancer.

### Identification of ceRNAs subnetwork with regard to overall survival in LC

**Figure 5:**
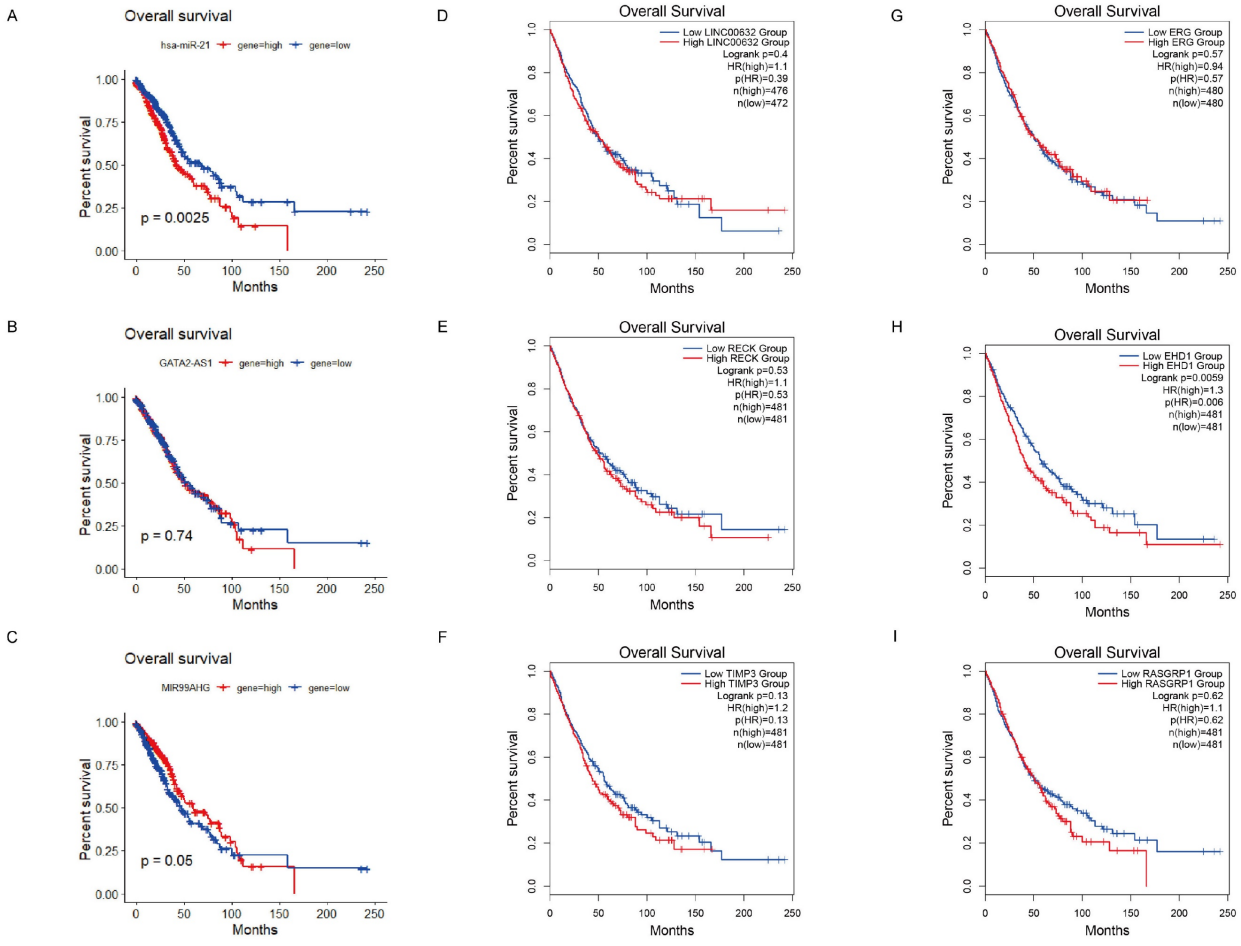
** Kaplan-Meier survival curves for the ceRNAs subnetwork.** (A) Overall survival analysis of TCGA cases based on hsa-miR-21-5p (*P* = 0.0025). According to the median expression level, the samples were divided into high expression group (red) and low expression group (blue). The horizontal axis is survival time, and the vertical axis represents the survival rate for the corresponding survival time. (B-D) Overall survival analysis of TCGA cases based on GATA2-AS1, MIR99AHG and LINC00632 (*P* = 0.74, 0.05, 0.4). (E-I) Survival analysis of 5 targeted genes (*RECK*, *TIMP3*, *EHD1*, *RASGRP1* and *ERG*) via GEPIA2. We selected clinical data from LUAD and LUSC patients for survival analysis. A significant correlation between low expression of *EHD1* and poor OS in LC patients (*P* = 0.0059).

According to the ceRNA hypothesis, we narrowed the primary lncRNA-miRNA-mRNA regulatory network, 3 downregulated lncRNAs (GATA2-AS1, LINC00632 and MIR99AHG), 1 miRNA (hsa-miR-21-5p) and 5 targeted genes (*RECK*, *TIMP3*, *EHD1*, *RASGRP1* and *ERG*) were selected as the ceRNA subnetwork. To further probe into the prognostic value of these RNAs, we conducted the Kaplan-Meier analysis in LC patients. As a result, lower expression of lncRNA MIR99AHG was found to be associated with worse OS (Figure 5C), and for the better OS when the expression level of hsa-miR-21-5p was lower (Figure 5A). Similarly, there is a significant correlation between low expression of *EHD1* and poor OS in LC patients, while the other targeted mRNAs represented no significant differences (Figure 5E-I). Notably, we discovered the interaction between lncRNAs and mRNAs of the ceRNAs, indicating that the interplay relationship is complex in the lncRNA-miRNA-mRNA regulatory network. To better understand the complexity of ceRNA regulatory network, we predicted the lncRNA-mRNA interactions using LncRRIsearch and found the possibility of direct pair binding between MIR99AHG and *EHD1*, which was hardly mentioned in previous studies (Table S6). MIR99AHG-hsa-miR-21-5p-*EHD1* was finally identified as a vital regulatory factor in the network, offering the potential for serving as biomarkers for predicting prognosis of LC (Figure 4D).

### Verification of DEM, DEL and DEGs

**Figure 6:**
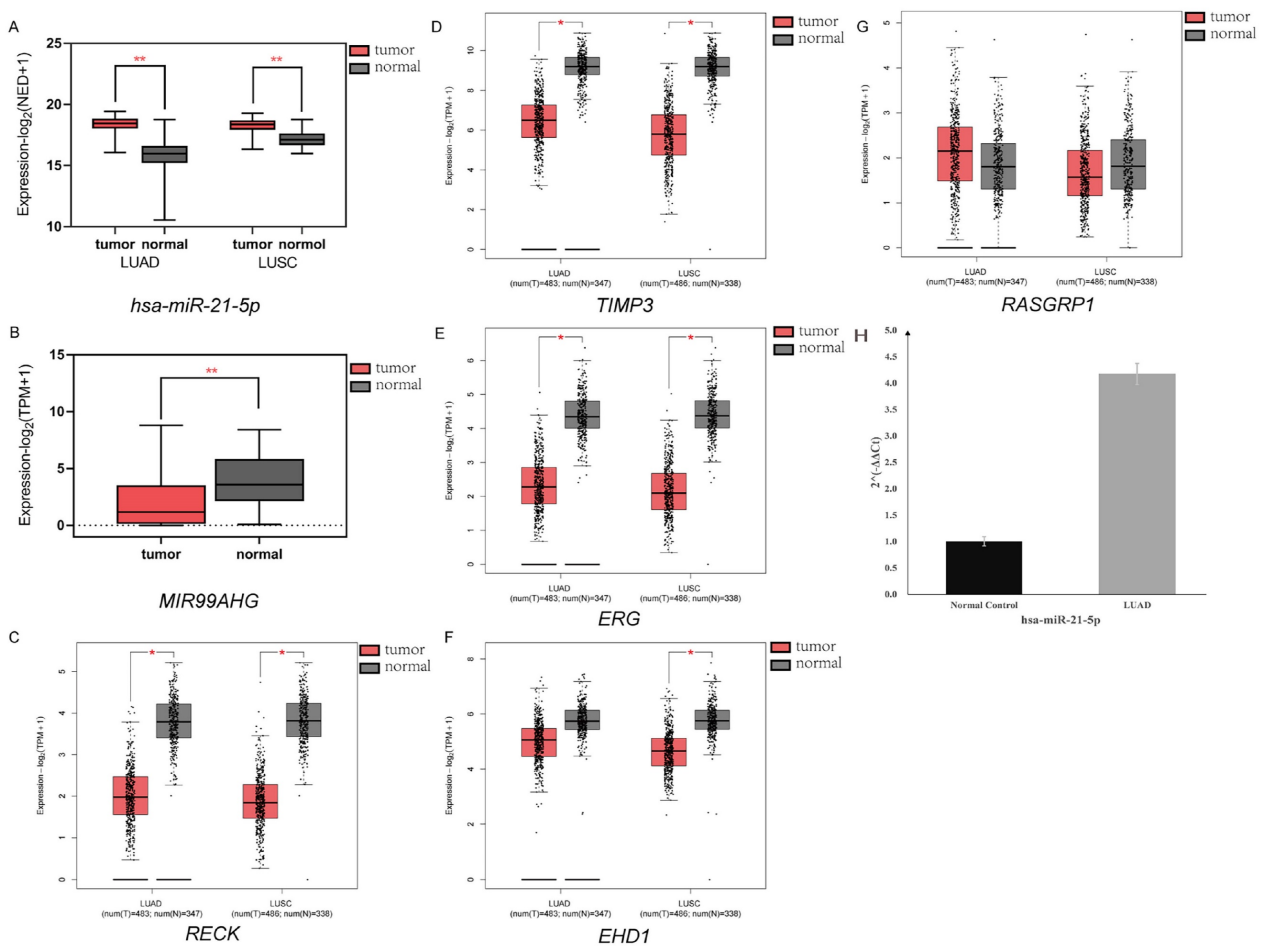
** Verification of selected DEM, DEL and DEGs.** (A) Expression of hsa-miR-21-5p via OMCD. Has-miR-21-5p was significantly highly expressed in both LUAD and LUSC patient samples, agreeing to our results. (*P* < 0.01). (B) Expression of MIR99AHG via LncExpDB. The results showed a low expression in LC (*P <* 0.01). (C-G) Expression of *RECK*, *TIMP3*, *ERG*, *EHD1* and *RASGRP1* via GEPIA2. The box plots showed that four genes (*RECK*, *TIMP3*, *ERG* and *EHD1*) were differentially expressed (*P* < 0.01, |log2FC| > 1) in at least one of the two lung cancers. (H) Expression of hsa-miR-21-5p via RT-qPCR. The results of RT-qPCR experiment also proved that the has-miR-21-5p expression in lung cancer tissues was significantly high (*P* < 0.01).

To verify the selected DEM (hsa-miR-21-5p), DEL (MIR99AHG) and 5 DEGs (*RECK*, *TIMP3, EHD1*, *ERG* and *RASGRP1*), we collected sequencing data from several cancer and normal tissues to explore the expression levels of these RNAs. The hsa-miR-21-5p expression levels in LC and normal lung tissue were analyzed via OMCD, which supplements 458 LUAD and 46 normal datasets, 343 LUSC and 45 normal datasets respectively. The two types account for an estimated 85% of all lung cancers. The normalized expression data, statistical results, and annotation data retrieved from OMCD were shown in Table S7. Hsa-miR-21-5p was overexpressed in LC specimens, agreeing to our results (Figure 6A, *P* < 0.01). For MIR99AHG validation, the raw data were obtained from LncExpDB and then developed the statistical analysis, the results showed a low expression in LC (Figure 6B,* P* < 0.01). GEPIA2 was used to verify the differential expression levels of *RECK*, *TIMP3*, *ERG*, *EHD1* and *RASGRP1*. The box plots generated by GEPIA2 showed that four genes (*RECK*, *TIMP3*, *ERG* and *EHD1)* were differentially expressed under the pre-set |log2FC| value and *P* value thresholds (Figure 6C-6G, *P* < 0.01) in at least one of the two lung cancers, with the exception of *RASGRP1*. The results of our RT-qPCR experiment also proved that the has-miR-21-5p expression in lung cancer tissues was significantly high (Figure 6H) (*P* < 0.01), and the specific expression information was shown in Table S8. These results proved that the selected RNAs were differentially expressed in lung cancer, demonstrating the reliability of our analysis results.

## Discussion

**Figure 7:**
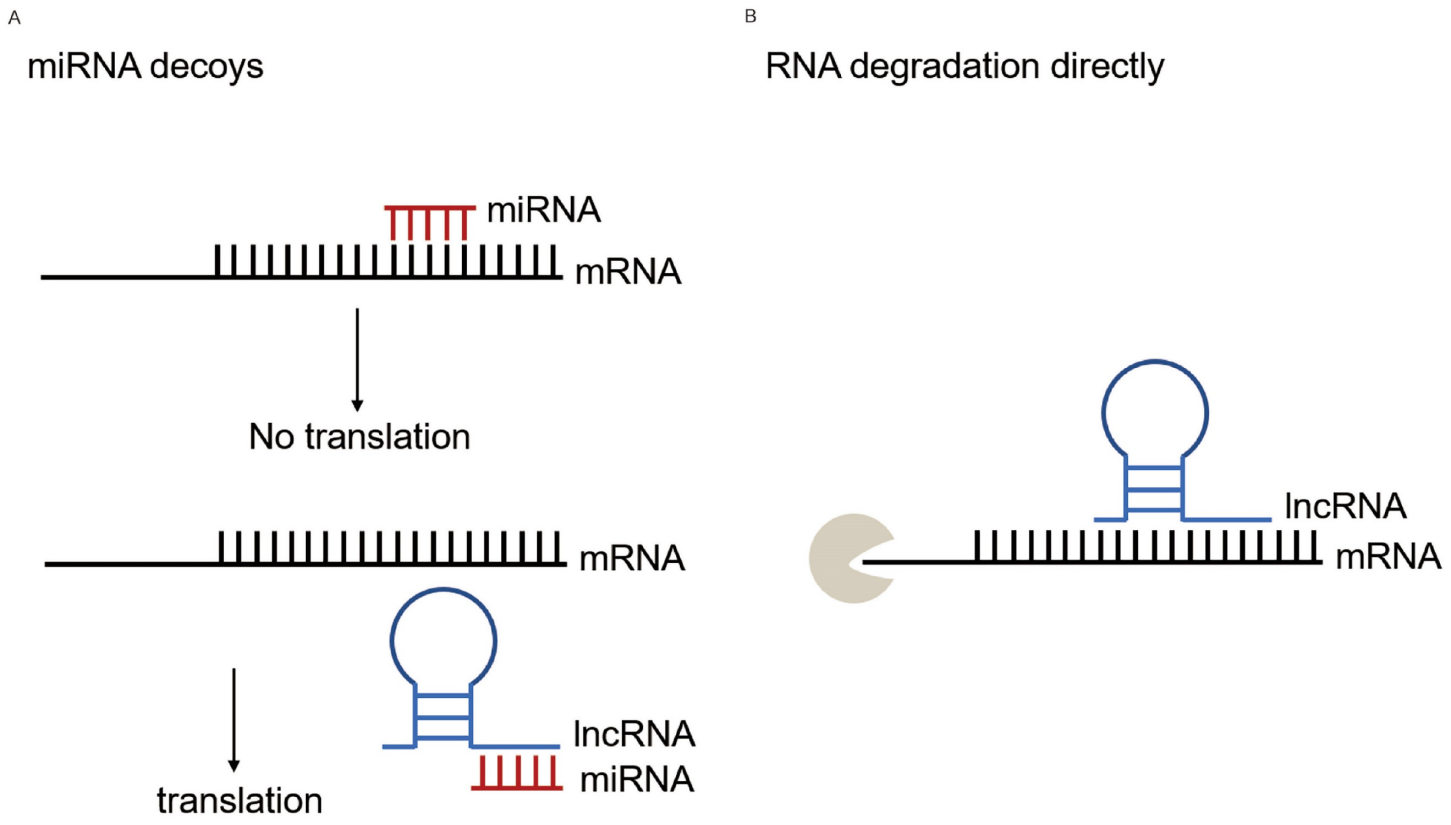
** Overview of lncRNA-miRNA-mRNA interplay.** (A) MiRNAs block translation or cause mRNA degradation by binding to it and lncRNAs indirectly regulate translation serving as miRNA decoys. In this study, the lower expression of lncRNA MIR99AHG in lung cancer patients resulted in more has-miR-21-5p binding to mRNA *EHD1*, thus causing its degradation or affecting its translation process at the post-transcriptional level. (B) LncRNAs directly regulate mRNA translation. Through LncRRIsearch prediction, MIR99AHG had a direct regulation with *EHD1*, besides the indirect effect through hsa-miR-21-5p.

With the innovation of high-throughput sequencing and bioinformatic analysis technologies, accumulating studies have characterized the potential role of lncRNA in tumorigenesis. As one of the representative examples of ncRNA, lncRNAs perform various functions in vivo and vitro, and have the potential of serving as biomarkers for the diagnosis of LC. The lncRNAs interplay with DNAs, RNAs and proteins, regulating various biological processes in chromatin, transcriptional and post-transcriptional levels 26. Particularly, lncRNAs located in the cytoplasm can affect mRNA expression by ceRNAs regulatory mechanism. In general, the miRNA-mRNA interactions have been elucidated that mature miRNAs bind to microRNA recognition elements (MRE) on the targeted mRNAs in the 3'and 5'untranslated regions (3'UTR and 5'UTR) by completely or non-completely base-paring rules 6. LncRNAs can play an effect on this process. Most studies described lncRNAs serving as miRNA sponges that attenuate the effects of miRNAs on mRNAs, in accordance with the ceRNAs hypothesis. LncRNAs, harboring similar miRNA target sequences, competitively bind to miRNAs and keep miRNAs away from binding with mRNAs, and the dis-regulation of mRNAs could generate a series of disease 13. For instance, Cheng et al. found that in acute myeloid leukemia (AML), the hub ceRNA network containing 6 circRNAs, 32 lncRNAs, 8 miRNAs and 6 mRNAs played an effect in the post-transcriptional regulatory mechanism 27. Ma et al. proved that LUAD-related networks, hsa_circ_0008234/hsa-miR-490-3p-*SYT1* and hsa_circ_0002360/hsa-miR-1293-*ADCY9*-*NMUR1*, involved in pathogenesis and prognosis in patients with LUAD 28. Besides indirectly operating at mRNAs, lncRNAs can aim at mRNAs directly (Figure 7). For example, the lncRNA, half-STAU1-binding site RNAs (1/2-sbsRNAs), form a binding site by incompletely base-paired with the Alu element in the 3'UTR of SMD targets, resulting in targeted mRNA decay 18. In summary, lncRNAs, miRNAs, or their combinations, play an essential role in DNA: RNA: protein interactions.

In this study, we found that MIR99AHG could emerge as a tumor regulator to play a role in malignant progression via hsa-miR-21-5p-*EHD1* axis in LC, while it also had direct base-pairing interaction with *EHD1*. The lncRNA that we identified, MIR99AHG, was identified as a long non-coding RNA located on chromosome 21q21.1. Some studies found that down-regulation of MIR99AHG was associated with lung adenocarcinoma, colorectal cancer, pancreatic cancer and breast cancer 22, 29-32. Meng et al found that MIR99AHG functioned as a miRNA sponge and negatively targeted miR-577.

In recent years, the biological function of miRNA is gradually being revealed. They serve as biomarkers alone or combine with others to support an early diagnosis and predict the prognosis of various cancers, including colorectal cancer (CRC) 33 and gastric cancer 34. Here, we found that hsa-miR-21-5p had an interaction with lncRNA MIR99AHG and *EHD1* in lung cancer. Previous studies had elucidated the oncogenic function of miR-21 in cancers. Wang et al reported that high expression of hsa-miR-21-5p in lung adenocarcinoma (LUAD) led to LUAD cell proliferation, migration and invasion through targeting WWC2 35. Jiang et al found that overexpression of miR-21-5p prompted inflammatory factors to release and activated pyroptosis via directly downregulating *TGFBI* in CRC 36.

Here, we analyzed five mRNAs, including *RECK*, *TIMP3*, *EHD1*,* RASGRP1* and *ERG* as the targeted genes of hsa-miR-21-5p. We found that MIR99AHG had a direct regulation with *EHD1*, besides the indirect effect through hsa-miR-21-5p. It is worth noting that the interaction between MIR99AHG and *EHD1* had not been reported in LC. Previous literature supports the roles of these five molecules in LC. *RECK* is a tumor suppressor gene, inhibiting angiogenesis, invasion, tumor metastasis and malignant development 37, 38. Guo et al described the roles of the miR-221/222-*RECK*-Notch1 axis in regulating cancer stem cells in non-small cell lung cancer (NSCLC) 39. *RECK* suppression via the STAT3/miR-92a axis promotes the invasiveness of lung cancer cells 40. However, the systematic analysis of the lncRNA-miRNA-mRNA network related to *RECK* in LC has not been explored. Tissue inhibitor of metalloproteinase 3 (*TIMP3*), one of the four members of the TIMP family, acts as a major regulator of the matrix metalloproteinases (MMPs) 41. *TIMP3* was proved to be a potential target of miRNAs, influencing downstream signal pathways such as proliferation, invasion survival and tumor growth in various cancers, including lung cancer 42, CRC 43 and breast cancer 44. In this research, we verified that the expression of *TIMP3* was significantly down-regulated in LC tissues compared with the normal tissues, which is identical to the theory that the silencing of *TIMP3* is consistently associated with cancer progression or poor patient prognosis. *EHD1*, a protein of the C-terminal Eps15 homology domain-containing family, participates in regulating endocytic recycling. Recent studies have found that *EHD1* played a key role in tumor development and progression of breast cancer and ovarian cancer 45, 46, including lung cancer 47. For instance, Liu et identified *EHD1* as a significant endocytic and metastasis-associated gene which enhanced the cancer stem cells-like properties, epithelial-mesenchymal transition and metastasis of LUAD cells by antagonizing the Hippo pathway 48. *RASGRP1*, a Ras guanine nucleotide exchange factor (RasGEF), is a key protein regulating effector kinases upon T cell antigen receptor (TCR) signaling 49. *RASGRP1* gene deficiency is associated with immune deficiencies 50. As a bifunctional regulator that promotes acute inflammation and inhibits inflammation-associated cancer, *RASGRP1* overexpression may suppress cancer cell growth and lead to a better prognosis in cancer patients 51. Several studies have also demonstrated that *RASGRP1* regulated cell proliferation and could be a novel therapeutic target via influencing other pathways in leukemia 52, hepatocellular carcinoma (HCC) 53 and CRC 54. The role of *RASGRP1* in lung cancer has not been shown. The ETS-related gene, belonging to the transcription factor ETS family, has been reported to involve in regulating cell proliferation, differentiation, migration, angiogenesis and tumorigenesis, including leukemia, cervical cancer and prostate cancer (PCa) 55, 56. Our results indicated the regulation network of *ERG* in lung cancer.

## Conclusion

In conclusion, we introduced a MIR99AHG-hsa-miR-21-5p-*EHD1* network in lung cancer bases on our high-throughput sequencing data, following various bioinformatics analyses. Firstly, we selected the DEMs, DELs and DEGs, then the lncRNA-miRNA pairs and miRNA-mRNA pairs were identified. We identified the lncRNA-miRNA-mRNA by integrating the common nodes. The only miRNA, hsa-miR-21-5p, and its crosstalk network were constructed, including 3 DELs (GATA2-AS1, LINC00632 and MIR99AHG), 1 DEM (hsa-miR-21-5p) and 5 DEGs (*RECK*, *TIMP3*, *ERG*, *EHD1* and *RASGRP1*). Furthermore, we analyzed the interplay between lncRNAs and mRNAs via LncRRIsearch and found that MIR99AHG had a direct regulation with *EHD1*, which had not been reported in LC. After Kaplan-Meier analysis, MIR99AHG-hsa-miR-21-5p-*EHD1* was finally identified as a vital regulatory subnetwork. We analyzed the expression levels of selected DEM, DELs, and DEGs in lung cancer patients and healthy people to verify our findings. As the key of ceRNA regulatory network, the expression of miR-21-5p in lung cancer patients was significantly higher than that in healthy people (*P* < 0.01), and its high expression was significantly associated with poor prognosis (*P* = 0.0025). On the flip side, the limitations of this study should be acknowledged. We did not perform further in vivo and in vitro experiments to identify the MIR99AHG-hsa-miR-21-5p-*EHD1* interaction network. Future investigations are necessary to validate our results in a larger cohort of lung cancer patients.

## Supplementary Material

**Table S1** Differentially expressed mRNAs (DEGs); **Table S2** Differentially expressed miRNAs (DEMs); **Table S3** Differentially expressed lncRNAs (DELs); **Table S4** Selected miRNA and targeted lncRNAs and genes; **Table S5** DEM-targeted genes; **Table S6** Down-regulated lncRNA-mRNA interaction via LncRRIsearch; **Table S7** The expression data of hsa-miR-21-5p in lung cancer; **Table S8** Primer sequences and experimental results of RT-qPCR.

## Abbreviations

LC: lung cancer
lncRNA: long non-coding RNA
miRNA: microRNA
ceRNA: competing endogenous RNA
DEG: differentially expressed gene
DEM: differentially expressed miRNA
DEL: differentially expressed lncRNA
NSCLC: non-small cell lung cancer
SCLC: small cell lung cancer
3'UTR: 3'untranslated region
LUAD: lung adenocarcinoma
TCGA: The Cancer Genome Atlas
OS: overall survival
OMCD: OncoMir Cancer Database
LUSC: lung squamous cell carcinoma
GEPIA2: Gene Expression Profiling Interactive Analysis 2
GTEx: Genotype-Tissue Expression
GO: Gene Ontology
MF: molecular function
CC: cellular component
BP: biological process
KEGG: Kyoto Encyclopedia of Genes and Genomes
RT-qPCR: quantitative reverse transcription polymerase chain reaction
MRE: microRNA recognition element
5'UTR: 5' untranslated regions
AML: acute myeloid leukemia
CRC: colorectal cancer
TIMP3: tissue inhibitor of metalloproteinase 3
MMP: matrix metalloproteinas
RasGEF: ras guanine nucleotide exchange factor
TCR: T cell antigen receptor
HCC: hepatocellular carcinoma
PCa: prostate cancer
